# The stand-alone Ayurveda treatment for the management of COVID-associated mucormycosis (CAM): a case report

**DOI:** 10.3389/fmed.2026.1848849

**Published:** 2026-06-05

**Authors:** Rajnikant Patel, Skanthesh Lakshmanan, Vinayak Padikkal, Keyur Mandaliya

**Affiliations:** 1 Agnivesh Ayurved Hospital, Surat, Gujarat, India; 2 Center for Advanced Studies and Integrated Research in Ayurveda, Thrissur, Kerala, India; 3 Clinical Research and Ethics Division, CASIRA Trust, Kerala, India; 4 Prismaa Oncoimaging, Surat, Gujarat, India

**Keywords:** Ayurveda, case report, emergency care, invasive fungai infection, mucormycosis

## Abstract

Mucormycosis is a rapidly progressive opportunistic fungal infection predominantly affecting individuals with diabetes or immune compromise. To our knowledge, this report presents one of the first clinically documented cases of invasive Mucormycosis managed exclusively with an Ayurveda-based approach. A 60-year-old male with recent COVID-19 infection (CT severity score 7/25) presented with facial heaviness, lacrimation, and peripheral vision loss. Diagnostic evaluation confirmed invasive mucormycosis through potassium hydroxide testing, histopathology demonstrating vasculoinvasion, fungal culture identifying Mucor species, and radiological findings. Surgical intervention including eye enucleation was advised. Following informed decision-making, the patient opted for stand-alone Ayurvedic management. A structured therapeutic regimen was administered over 15 days and continued for 90 days. The patient demonstrated progressive symptomatic improvement with no reported adverse events. At the three-year follow-up, there was no evidence of clinical recurrence. Laboratory parameters, including liver and renal function, remained within normal limits. Follow-up imaging indicated resolution with residual structural changes. This case documents a rare and complex clinical scenario managed with an Ayurveda-based approach, demonstrating sustained clinical and radiological improvement. While the findings do not permit generalisation, they highlight a state of clinical equipoise and underscore the need for rigorously designed studies to systematically evaluate the safety, effectiveness, and potential role of Ayurveda-based interventions in the management of invasive mucormycosis.

## 1 Introduction

Mucormycosis is a rare but rapidly progressive opportunistic angioinvasive fungal infection caused by filamentous fungi belonging to the order *Mucorales.* The disease predominantly affects immunocompromised individuals and patients with uncontrolled diabetes mellitus, corticosteroid exposure, haematological malignancies, or COVID-19–associated immune dysregulation (1). Clinically, rhino-orbital mucormycosis (ROM) represents the most common and severe presentation, characterised by rapid invasion of the nasal cavity and paranasal sinuses with potential extension into the orbit and intracranial structures through angioinvasion and tissue necrosis (2). The global prevalence of ROM estimates ranging from 0.0005 to 0.17% (0.005–1.7 cases per million population) (3). Asia contributes nearly 31% of reported global cases, while India exhibits the highest documented burden at approximately 0.014% (140 cases per million population), nearly 82-fold higher than global estimates (4). The high burden in India has been associated with factors such as diabetes mellitus, corticosteroid exposure, and environmental conditions favourable for fungal proliferation (4). ROM manifestation associated with the COVID-19 condition is referred to as COVID-associated mucormycosis (CAM). CAM possesses a significant mortality rate between 23% and 38% and also possesses debilitating morbidities affecting the eyes, brain functioning, and facial disfigurement (5, 6). ROM has been observed to have an association with a recent history of COVID-19, and potential pathophysiological connections have also been proposed (7). ROM management requires critical care due to its tissue-destructive nature and quick progression (8). The standard of care (SOC) for the management of ROM comprises surgical debridement and antifungal drugs. Studies have observed a significant mortality rate in patients of ROM even after SOC treatment and reported the recurrence of the disease (9). Antifungal therapeutic agents are observed to have side effects, including nephrotoxicity (10). The surgical debridement is often observed to produce disfigurement, which also causes overall distress to patients (11). In this context, it is important to explore potential strategies that can enhance overall treatment outcomes and minimise the potential risk for ROM management. Ayurveda, an Indian traditional system of medicine has significant preference among the population around India and the subcontinent. Ayurveda offers different levels of interventions, such as internal medications, external therapies, diet, and lifestyle guidelines. There is limited documented scientific evidence of the effectiveness of Ayurveda formulations in managing Mucormycosis.

However, the authors present a novel case of a CAM patient managed solely with Ayurveda, possibly the first such report to our knowledge.

## 2 Case history

A 60-year-old male patient from India approached the first author with symptoms of heaviness on both sides of the nose and eyes, forehead, and upper palate. Additionally, the patient had profuse lacrimation from the right eye, double vision and progressive loss of peripheral vision in the same eye that appeared over 10 to 12 days. The patient’s family history revealed the absence of any particular diseases, especially diabetes, hypertension and cancer. The patient had a history of COVID-19 infection, diagnosed on 9th April 2021. Then a High-Resolution Computed Tomography (HRCT) scan revealed 15% parenchymal lung involvement with a CT severity index of 7/25. He had no history of diabetes, hypertension, or immunosuppressive treatment. The patient has also not reported systemic dysfunctions like renal and hepatic dysfunction. The patient received biomedical treatment for COVID-19 and recovered. However, approximately 2 weeks after recovery, he developed localised facial and ocular symptoms.

### 2.1 Diagnosis

In this case, the patient encountered multiple challenges, including limited access to diagnostic testing and significant financial and cultural stigma associated with the condition. These difficulties were further exacerbated by the COVID-19 pandemic, which intensified existing barriers to diagnosis.

The patient initially consulted a biomedical ENT surgeon prior to Ayurveda consultation. Potassium Hydroxide (KOH) wet mount examination of tissue obtained from the right sino-nasal region demonstrated fungal elements characterised by few broad, aseptate, branching fungal hyphae, suggestive of mucormycosis. Histopathological examination of endoscopic tissue sampled from the right-sided sinus revealed areas of necrosis with tissue-invasive and angioinvasive broad aseptate fungal hyphae, along with associated acute inflammatory changes, findings consistent with mucormycosis. No granuloma or malignancy was identified. Fungal culture of the tissue specimen further demonstrated growth of *Mucor* spp. based on microbiological evaluation. Molecular species-level identification and antifungal susceptibility testing were not performed due to resource limitations.

Computed Tomography of the Paranasal Sinuses (CT PNS) and brain imaging revealed bilateral nasal bone involvement, right-sided pan-rhinosinusitis with bone erosions and right orbital cellulitis, which are indicative of CAM (Figure 1). (All these evaluations were done prior to the patient approaching the first author for Ayurveda treatment). The diagnosis of CAM was established by integrating the patient’s history of COVID-19, clinical features, KOH examination, histopathology, fungal culture, and CT imaging, with differentiation from invasive aspergillosis and bacterial rhinosinusitis.

**Figure 1:**
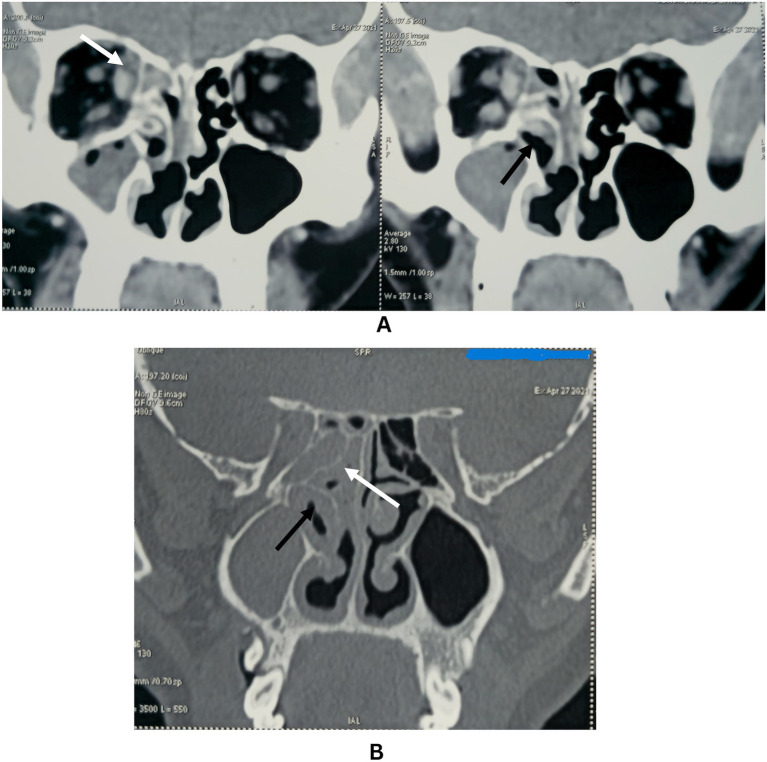
CT image before Ayurveda treatment. This figure contains two panels of coronal CT images **(A,B)** of the paranasal sinus and orbit obtained before initiation of Ayurveda treatment, demonstrating right-sided sino-orbital inflammatory involvement. **(A)** White arrow indicates ill-defined enhancing soft tissue along the right medial rectus muscle with associated enhancement of adjacent muscle fibres compared to the contralateral side, suggestive of orbital inflammatory involvement. Black arrow indicates enhancing soft tissue involving the right infundibulum/medial meatus region, corresponding to histopathologically confirmed fungal inflammatory soft tissue. **(B)** White arrow demonstrates rarefaction of the right ethmoid lamina papyracea with adjacent soft tissue fullness in the superior nasal cavity. Black arrow indicates rarefaction of the right middle turbinate with associated soft tissue fullness involving the infundibulum, right osteomeatal complex (OMC), and middle meatus region.

The primary consultant ENT doctor recommended right eye enucleation and Functional Endoscopic Sinus Surgery (FESS) as emergency care. However, perceiving the suggested biomedical treatment as relatively risky, the patient preferred Ayurveda and approached the first author through referrals from previously treated mucormycosis patients. Following a fair informed consent process and shared decision-making, the patient declined invasive intervention and opted for stand-alone Ayurvedic management. The Ayurveda medical team also informed the patient that his clinical condition would be closely monitored throughout treatment and that immediate referral to biomedical care would be initiated if his symptoms worsened, failed to improve, or if any clinical red-flag signs developed.

In approaching the first author, the physical examination revealed inflammation in the right side of the face, tenderness at right side of the cheeks and periorbital area.

### 2.2 Treatment

According to Ayurvedic clinical interpretation, the condition was correlated with *Raktaja Krimi* (pathological organisms affecting the blood and vascular tissues) and *Asthi-Majjagata Krimi* (pathological involvement of bone and marrow tissues), based on descriptions provided in the WHO International Standard Terminologies on Ayurveda (ITA 5.13.7; ITA 2.2.1 and 5.13) (12).

The treatment has been developed based on a comprehensive understanding of both CAM and relevant Ayurveda conditions described above. The therapeutic approach in this case comprised internal Ayurvedic formulations, external therapies including herbal fumigation and ophthalmic preparations, along with supportive dietary and lifestyle modifications. Internal interventions included tablet formulations, capsules, and herbal powders administered in different schedules according to the stage-wise clinical assessment of the patient. The dosage and frequency of administration varied across formulations and therapeutic phases. Table 1 provides a structured overview of the Ayurvedic treatment protocol, including formulation composition, dosage, administration schedule, duration, classical references, and therapeutic rationale (Table 1).

**Table 1:** Data on internal medicines and external therapy.

	Medicine name	Particulars	Dose	Duration (From–To)	Reference	Rationale
1.	Brihat vat chintamani rasa	Herbo mineral formulation containing purified Rasa (Purified Mercury), Gandhaka (Purified Sulfur), Abhraka Bhasma (Calcinated Mica)	(125 mg) 1 tab twice a day	27/04/2021–10/06/2021 (Total 45 days)	*Book-Bhaishajya Ratnavali, 26/145-148, Indicated in disease of Vata dosha (ITA-2.1.1.1 Vata, dosha regulating movement and cognition) and Rasayana (ITA-9.1.35 Rejuvenative therapy)* (10)	The formulation was used considering its indication for the management of the disease of Vata dosha (ITA-2.1.1.1 Vata, dosha regulating Movement, cognition) and its potential as Rasayana (ITA-9.1.35 Rejuvenative therapy) (10)
2.	Krumikuthar rasa	Herbo mineral Formulation containing Kajjali (Black Sulfide of Mercury), Tankana Bhasma (Borax)	(125 mg) 2 tab thrice a day	27/04/2021–21/06/2021 (Total 56 days)	Book - Ayurved sara Sangrah (Page no 323), Indicated in Krimi roga (Disease of Krimi, ITA-5.13.4 Internal worm Infestation)	The formulation was used considering its indication for the management of Krimi roga (Disease of Krimi, ITA-5.13.4 Internal worm Infestation)
3.	Krumighna vatika	Herbal Formulation containing Vidanga (*Embelia ribes*), Ajamoda (*Apium graveolens*), Haritaki (*Terminalia chebula*)	(250 mg) 2 tabs thrice a day	27/04/2021–25/07/2021 (Total 90 days)	Book- *Sahasrayogam* *Indicated in* Krimi roga (Disease of Krimi, ITA-5.13.4 Internal worm Infestation) (10)	The formulation was used considering its indication for the management of Krimi roga (Disease of Krimi, ITA-5.13.4 Internal worm Infestation) (10)
4.	Serenkottai nei capsule	Herbal combination containing Shuddha Bhallataka (*Semecarpus anacardium*), Milk, Ghee	1 capsule (10-15 ghee drops) thrice a day as a nasal drop (Half a capsule has to be instilled in both nostrils)	27/04/2021–25/07/2021 (Total 90 days)	Book- Siddha Vaithya Thirattu Indicated in Kapha vyadhi (disease of Kapha, ITA-2.1.3.1 Kapha, dosha responsible for cohesiveness) (10)	The formulation was used considering its indication for the management of Kapha vyadhi (disease of Kapha, ITA-2.1.3.1 Kapha, dosha responsible for cohesiveness) (10)
5.	Asthiposhak vati	Herbal formulation containing Kukkudantatwak Bhasma (Egg shell calx), Asthishringala (*Cissus Quadrangularis*), Arjuna (*Terminalia arjuna*), Laksha (*Laccifer Lacca*), Amalaki (*Phyllanthus emblica*), Ashwangandha (*Withania Somnifera*), Guduchi (*Tinospora Cordifolia*), Guggulu (*Commiphora mukul*), Bala (*Sida Cordifolia*), decoction of Babbula (*Acacia Arabica*)	250 mg 2 tabs twice a day	27/04/2021–02/07/2021 (Total 67 days)	Ayurveda Proprietary Medicine. Indicated to strengthen the bony tissue	The formulation was used considering its indication to strengthen the bony tissue
6.	Vidang choorna	Herbal Powder contains Vidanga (*Embelia ribes*), Amla (*Indian Gooseberry*)	(5–10 gm) To drink and eat food with vidang choorna added into it	27/04/2021-25/07/2021 (Total 90 days)	Book- Bhava Prakash Indicated as the Best herb for Krimi (ITA-5.13.4 Internal worm Infestation) (10)	The formulation was used considering its superior first-line indication for the management of Krimi roga (Disease of Krimi, ITA-5.13.4 Internal worm Infestation) (10)
7.	Palash choorna 400 gram + Bhallatak choorna 50 gram + Vilwadi gulika 100 tablets + Vidang choorna 100 gram	Herbal Formulations contains powder of Palash (*Butea monosperma*), Bhallathaka (*Semecarpus anacardium*), Vilwa (*Aegle marmelos*), Vidanga (*Embelia ribes*)	Mix all together and the patient has to inhale and exhale the smoke generated in it morning and in the evening	27/04/2021–25/07/2021 (Total 90 day)	Palash choorna Book- Bhava Prakash Indicated as the Best herb for Krimi (ITA-5.13.4 Internal worm Infestation), Bhallatak choorna (Indicated in Kapha vyadhi disease of Kapha, ITA-2.1.3.1 Kapha, dosha responsible for cohesiveness) Vilwadi Gulika, Book – Ashtanghridayam, indicated in visha (TA-5.72.1 Poisoning) (10)	The combination was used considering its multiple indications. Palash choorna, superior-first line indication for the management of Krimi (ITA-5.13.4 Internal worm Infestation), Bhallatak choorna as indicated (Indicated in Kapha vyadhi (disease of Kapha, ITA-2.1.3.1 Kapha, dosha responsible for cohesiveness). Vilwadi Gulika as indicated for the management of visha (TA-5.72.1 Poisoning) (10)
8	Netra Sudarshan Ark	Eye drop containing Palash (*Butea monosperma*)	2 eye drops in affected eye 3 times a day	27/04/2021–25/07/2021 (Total 90 day)	*Book-* Ras Tantra Saar & Siddha Prayog Sangrah *Volume 1, Indicated in netra roga (ITA-5.66.1 Eye disorders)* (10)	*The formulation was used considering its indication for the management of Netra roga (ITA-5.66.1 Eye disorders)* (10)

The Ayurvedic medicines administered in this case adhered to established standards of quality and safety. All formulations were produced under Good Manufacturing Practice (GMP)-certified facilities and were duly approved and regulated by the Central and State Licensing Authorities of AYUSH, as well as the Drug Control Department (Table 2).

**Table 2:** Information on follow-up and outcome.

Day	Follow-up	Progress
15 days	First follow-up	70% improvement in heaviness over nose, eyes, and upper palate 50% improvement in heaviness of the upper palate 90% reduction in lacrimation
45 days	Second follow-up	Complete resolution of heaviness in the nose, eyes, and upper palate, Marked reduction in lacrimation and approximately 60% improvement in peripheral vision
80 days	Third follow-up	Substantial improvement in peripheral vision, reported at around 95%
3 Months	Fourth follow-up	Completely asymptomatic, with full resolution of all previously reported symptoms.
3 Years	Long-term follow-up	Imaging showed healed right-sided rhinosinusitis Residual sclerosis of the bony walls of the right sinuses and orbit Healed/granulation tissue seen in the right maxillary and sphenoid sinuses, indicating stable recovery

The patient was instructed to take easily digestible and kleda (moist ness/soddening—ITA- D.2534) reducing foods and include camel milk, and consumption of bitter vegetables and adopt a lifestyle which reduces the kleda (moist ness/soddening—ITA- D.2534) (10). The patient was advised to adopt an easily digestible diet and lifestyle regimen intended to reduce *kleda* (a concept referring to excess moisture or pathological fluid accumulation; ITA-D.2534), which included the consumption of camel milk and bitter vegetables (12).

To reduce kleda (moist ness/soddening), lifestyle and diet should minimize cold, heavy, and moistening factors. Avoid daytime sleep, excess sweet and sour tastes, prolonged air-conditioning, cold breeze exposure, and most dairy except camel milk. Promote warmth and dryness via sunlight, bitter and pungent tastes, and moderate camel milk intake. These measures aid kleda regulation and restore metabolic balance.

### 2.3 Outcome and follow up

The patient has shown consistent clinical progress during the regular follow-ups after 15 days.

Day of initiating Ayurveda treatment. The CT scan 3 years post-treatment shows no active CAM with complete healing of right-sided sinusitis of the granulation tissue in the right maxillary and sphenoid sinus. Residual sclerosis of the bony walls of the right-sided sinus and orbit (Figure 2). Intervention adherence and tolerability were assessed clinically through consistent treatment uptake, absence of reported adverse events, and patient-reported satisfaction during follow-up consultations, yielding complete compliance. Also, the patient has not reported any side effects during the Ayurveda treatment. A blood test conducted after 3 years of follow-up (on 13 October 2025) confirmed normal liver and kidney function values.

**Figure 2:**
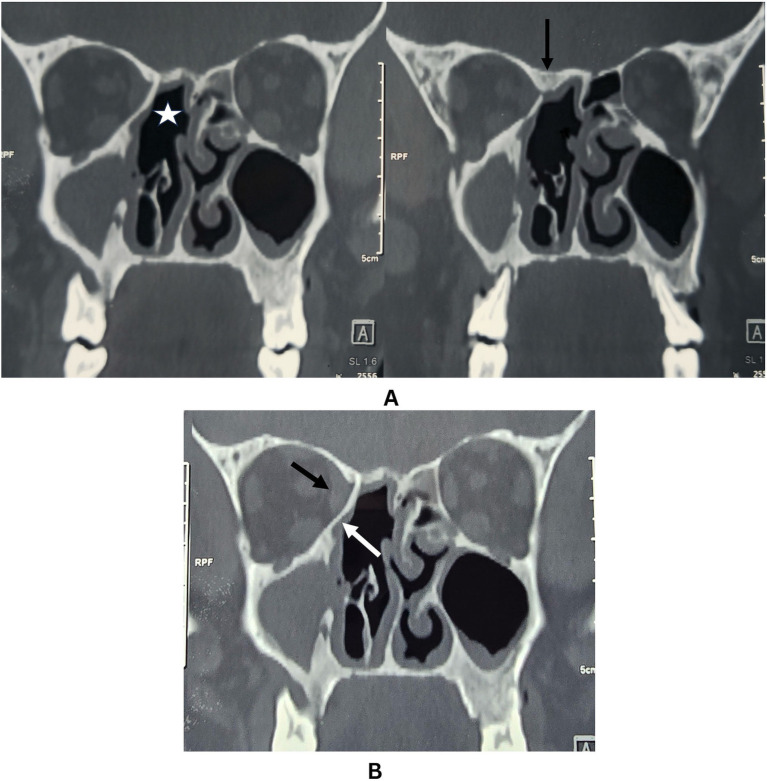
CT image after Ayurveda treatment. This figure contains two panels of coronal CT images **(A,B)** of the paranasal sinus and orbit obtained after Ayurveda treatment, demonstrating interval radiological changes. **(A)** Black arrow indicates interval resolution of the previously noted soft tissue thickening along the right medial rectus region. White star demonstrates subtle interval sclerosis of the right lamina papyracea. **(B)** The white arrow indicates near-complete resolution of the previously visualized soft tissue involvement within the right middle meatus and ethmoid air cells, with improved pneumatisation of the right nasal cavity. Black arrow demonstrates interval subtle sclerosis involving the horizontal aspect of the right ethmoid bone.

## 3 Discussion

In this case, the authors report successful management of CAM using stand-alone Ayurveda treatment. Resolution of infection on CT, along with the patient’s symptom-free status after 3 years (Day +1,096) of follow-up, suggests a favourable clinical outcome.

Histopathological evidence of tissue-invasive fungal hyphae, culture confirmation of Mucor species from sterile biopsy, and radiological features of invasion collectively fulfil the EORTC/MSGERC European Organisation for Research and Treatment of Cancer(EORTC) / Mycoses Study Group Education and Research Consortium (MSGERC) criteria for proven invasive fungal disease in this case (13).

Earlier studies report that primary ROM symptoms are nasal-sinus manifestations such as facial pain, nasal blockage, and inflammation. Progression to ocular symptoms reflects disease advancement with orbital infiltration and optic nerve involvement (14). Here, the patient initially consulted an ENT surgeon with nasal-sinus symptoms; approximately 10 to 12 days before to initiation of the Ayurveda treatment. At the first Ayurvedic consultation the predominant symptoms were ocular, including diminished vision. This progression indicated that initiation of Ayurveda treatment in this case occurred during an advanced phase of disease manifestation. SOC for advanced ROM typically includes systemic antifungal therapy, surgical debridement like FESS, and orbital exenteration. An observational study reported a 44.2% mortality rate among ROM patients receiving SOC, often with multiple surgeries (14). In contrast, in this case, the patient received only Ayurveda treatment (Table 2) without any invasive procedures. Notably, over a three-year follow-up period (Day +1,096), the patient remained asymptomatic, with CT imaging confirming infection resolution achieved through non-invasive Ayurveda treatment. Management of advanced ROM often requires critical care (15). Uğurlu et al. (16) noted that treatment delays beyond 12 days reduce survival to 36%–42% and earlier scientific publications observed delay of treatment for more than 6 days reduces survival rate by almost half (17). Here, Ayurveda treatment was initiated approximately 12 days after the onset of initial symptoms, a period typically associated with poorer outcomes in conventional care. This highlights the need to examine Ayurveda’s potential when instituted during such a critical window of disease progression.

Amphotericin B and its intravenous formulations are associated with nephrotoxicity and infusion-related adverse effects, which pose significant clinical challenges (10). Additionally, surgical interventions carry risks such as nerve damage and facial disfigurement (17). In contrast, the patient in this case did not report any adverse effects from stand-alone Ayurveda treatment, thereby eliminating the potential distress associated with invasive surgery. Throughout treatment and follow-up, the patient was closely monitored by the treating physician for any adverse effects, none of which were reported in this case. Furthermore, a blood test conducted on 13 October 2025, after 3 years of follow-up, confirmed normal liver and kidney function, supporting the safety of the formulations used in this case. This suggests a favourable risk–benefit profile of Ayurveda treatment, particularly in light of the observed clinical outcomes. Herbo-mineral formulations constitute an important pharmaceutical category within Ayurveda and may contain processed metallic and mineral components prepared through classical purification and incineration procedures described in Ayurvedic pharmaceutics. Broader toxicological concerns regarding heavy metal exposure and associated adverse effects, including hepatotoxicity, nephrotoxicity, neurotoxicity, and gastrointestinal complications, have been discussed in contemporary toxicological and regulatory literature (18). Regulatory agencies, including the United States Food and Drug Administration (US-FDA), have also highlighted potential safety concerns associated with improperly manufactured or unsupervised use of such formulations (18). Some published studies have reported favourable safety observations or absence of major adverse events with certain herbo-mineral preparations manufactured according to classical Ayurvedic procedures and administered within regulated settings (19). Nevertheless, significant challenges remain regarding the establishment of universally accepted standardisation protocols, quality assurance systems, pharmacovigilance frameworks, and toxicity-reporting guidelines for Ayurvedic herbo-mineral medicines (20). These concerns underscore the importance of rigorous manufacturing standards, contaminant testing, and long-term safety evaluation in both clinical practice and research.

Previous scientific studies, including biocomputational analyses as well as *in vitro* and *in vivo* experiments, have reported potential antifungal properties of various Ayurvedic medicinal plants (21–23). A cytological study evaluating an Ayurvedic herbal nasal formulation demonstrated activity against *Mucor* spores mediated through TNF-α (24). In the present case, the most commonly used herbal drugs across formulations include Vidanga (*Embelia ribes*), Palash, Bhallataka (*Semecarpus anacardium*), Shirisha (*Albizia lebbeck*), and Neem (*Azadirachta indica*). Notably, embelin, a bioactive constituent of *Embelia ribes*, has been reported to inhibit TNF-*α* converting enzyme (TACE) and downregulate TNF-*α*–dependent NF-κB signalling in experimental models (25). Although the directionality of TNF-α modulation differs between embelin and the herbal nasal formulation, these experimental observations indicate a potential role for embelin-based constituents in modulating TNF-α–mediated pathways associated with infection and inflammation. Additionally, experimental evidence suggests antifungal activity of several drugs used in this case (26, 27). Collectively, the available evidence on antifungal, anti-inflammatory, and immunomodulatory properties may offer a plausible mechanistic basis for the observed clinical course in the management of CAM in this case. However, *in vivo* and *in vitro* experiments are essential to evaluate the specific action of these five herbs against mucormycosis and establish clear biological plausibility. Previous reports confirm that processed metal-based nanotherapeutics disrupt microbial cells by inducing oxidative stress, leading to cellular dysfunction and death. This evidence reflects the general mechanism of action for nanotherapeutic derivatives and plausibly extends to the specific herbo-mineral formulations employed in this case (28). Considering clinical evidence, Madikonda et al. (29), in a non-randomised clinical trial involving 92 participants, reported overall clinical and radiological improvement with Ayurveda as an adjunct therapy. The pattern of clinical and radiological improvement reported in that study is comparable to observations in the present case. However, the study involved a follow-up duration of 45 days with Ayurveda administered as an add-on therapy, whereas the present report describes a 3-year follow-up with Ayurveda as a standalone intervention. It is important to emphasise that this report is based on a single case and is not intended to support clinical advocacy. Rather, it is presented to contribute to hypothesis generation and to support clinical equipoise for evaluating Ayurveda-based interventions in the management of CAM through rigorously designed clinical trials assessing effectiveness and safety.

The patient stated in their own words after undergoing Ayurveda treatment, “We were having much doubt whether Ayurvedic treatment will save me from fungus or not, but I am happy to see the impact it has made on my illness and It also saved me many lakhs of rupees (hundreds of thousands of INR)” which reflected overall benefit the patient gained from Ayurveda treatment.

A limitation of this report is the absence of comprehensive objective ophthalmological assessments, including standardised visual acuity and perimetry evaluations during follow-up. In addition, post-treatment microbiological confirmation was not performed; therefore, complete fungal eradication could not be definitively established despite the observed clinical and radiological improvement. However, in real-world clinical practice, particularly within certain low- and middle-income countries (LMIC) settings, repeat microbiological testing following clinical stabilisation and radiological improvement may not always be routinely feasible or incorporated into long-term outcome assessment. Additionally, several major clinical studies evaluating mucormycosis outcomes have similarly relied predominantly on clinical and radiological parameters rather than post-treatment culture negativity as an explicit endpoint (30).

## 4 Conclusion

This case describes the clinical course of a patient with COVID-19-associated mucormycosis (CAM) managed using a stand-alone Ayurveda-based therapeutic approach comprising internal formulations, external therapies, and supportive dietary and lifestyle modifications. Significant clinical and radiological improvement was observed during the treatment course and throughout the 3-year follow-up period. However, given the inherent limitations of a single-case observation, definitive conclusions regarding therapeutic effectiveness or complete fungal eradication cannot be established. Accordingly, these findings should be interpreted as exploratory and hypothesis-generating. This case highlights the need for further rigorously designed clinical research to evaluate the safety, therapeutic potential, and broader applicability of Ayurveda-based interventions in the management of CAM.

## Data Availability

The original contributions presented in the study are included in the article/supplementary material, further inquiries can be directed to the corresponding author.
